# Control of Cellular Bcl-x_L_ Levels by Deamidation-Regulated Degradation

**DOI:** 10.1371/journal.pbio.1001588

**Published:** 2013-06-25

**Authors:** So Hee Dho, Benjamin E. Deverman, Carlo Lapid, Scott R. Manson, Lu Gan, Jacob J. Riehm, Rajeev Aurora, Ki-Sun Kwon, Steven J. Weintraub

**Affiliations:** 1 Division of Urology and The Alvin J. Siteman Cancer Center, Washington University School of Medicine, Saint Louis, Missouri, United States of America; 2 Laboratory of Cell Signaling, Aging Research Center, Korea Research Institute of Bioscience and Biotechnology, Yusong, Daejeon, Korea; 3 Department of Biology, Washington University, Saint Louis, Missouri, United States of America; 4 Department of Molecular Microbiology and Immunology, Saint Louis University School of Medicine, Saint Louis, Missouri, United States of America; 5 Department of Internal Medicine, Washington University School of Medicine, Saint Louis, Missouri, United States of America; 6 Department of Internal Medicine, St. Louis VA Medical Center–John Cochran Division, Saint Louis, Missouri, United States of America; St. Jude Children's Research Hospital, United States of America

## Abstract

The cellular concentration of Bcl-x_L_ is among the most important determinants of treatment response and overall prognosis in a broad range of tumors as well as an important determinant of the cellular response to several forms of tissue injury. We and others have previously shown that human Bcl-x_L_ undergoes deamidation at two asparaginyl residues and that DNA-damaging antineoplastic agents as well as other stimuli can increase the rate of deamidation. Deamidation results in the replacement of asparginyl residues with aspartyl or isoaspartyl residues. Thus deamidation, like phosphorylation, introduces a negative charge into proteins. Here we show that the level of human Bcl-x_L_ is constantly modulated by deamidation because deamidation, like phosphorylation in other proteins, activates a conditional PEST sequence to target Bcl-x_L_ for degradation. Additionally, we show that degradation of deamidated Bcl-x_L_ is mediated at least in part by calpain. Notably, we present sequence and biochemical data that suggest that deamidation has been conserved from the simplest extant metazoans through the human form of Bcl-x_L_, underscoring its importance in Bcl-x_L_ regulation. Our findings strongly suggest that deamidation-regulated Bcl-x_L_ degradation is an important component of the cellular rheostat that determines susceptibility to DNA-damaging agents and other death stimuli.

## Abstract 2

Deamidation of two asparagines activates a conditional PEST sequence to target Bcl-xL for degradation.

## Author Summary

Cellular levels of the pro-survival protein Bcl-x_L_ are an important determinant of cellular susceptibility to many death stimuli, including most cancer therapies. We previously showed that human Bcl-x_L_ undergoes deamidation – the conversion of two neutral asparaginyl side-chains into negatively charged aspartyl side-chains – a process that occurs spontaneously but is accelerated by the treatment of tumor cells with DNA-damaging agents. Here, we show that deamidation activates a hitherto undetected signal sequence within Bcl-x_L_ that targets it for degradation by a pathway involving the proteolytic enzyme calpain. This increased degradation of Bcl-x_L_, and the consequent enhanced cellular susceptibility to programmed cell death, may contribute to the ability of DNA-damaging agents to kill tumors. We also demonstrate that deamidation of Bcl-x_L_ has likely been conserved from the simplest metazoans to humans, underscoring the importance of deamidation in the regulation of Bcl-x_L_.

## Introduction

The Bcl-2 proteins are grouped into those that promote cell survival and those that promote programmed cell death [1]. It is thought that the balance of activity of these two groups of proteins serves as a rheostat that determines whether the cell lives or dies [2]. The activity of the prosurvival Bcl-2 proteins is normally dominant in a cell. Most antineoplastic agents and other proapoptotic agents induce changes in Bcl-2 proteins that tip the balance towards the prodeath activity [3]. Importantly, this may involve a decrease in the activity of prosurvival proteins, an increase in the activity of prodeath proteins, or a combination of both.

There is substantial evidence that the level of the prosurvival Bcl-2 family protein Bcl-x_L_ is one of the most important cellular determinants of patient outcome in a broad range of tumors. For example, increased Bcl-x_L_ expression portends a worse prognosis in pancreatic cancer [4], thyroid cancer [5], follicular lymphoma [6], ovarian cancer [7],[8], hepatocellular carcinoma [9], and prostate cancer [10] and it has been specifically shown that increased levels of Bcl-x_L_ correlate with treatment failure in thyroid cancer [5], ovarian cancer [8], and oropharyngeal cancer [11]. In support of a functional role for Bcl-x_L_ in determining the prognosis and treatment response of patients with these cancers are the findings that (i) there is a “striking” correlation between resistance to treatment with a panel of 122 chemotherapeutic agents and Bcl-x_L_ expression levels when assessed in 60 different types of tumor cells [12]; (ii) overexpression of Bcl-x_L_ confers a multidrug resistance phenotype to tumor cells [13]; (iii) a small molecule or antisense that selectively inhibits Bcl-x_L_ increases sensitivity to chemotherapy in vivo [14],[15]; (iv) at least in some cells, there is a *bcl-x* gene-dosage effect for resistance to DNA-damaging agents [16]; and (v) increased Bcl-x_L_ expression increases susceptibility to carcinogen-induced tumor formation in mice [17]. When considered together, these findings suggest that tumor cell Bcl-x_L_ levels have an important functional role in determining patient outcome.

The expression level of Bcl-x_L_ is also important in determining the extent of damage in certain forms of tissue injury; in fact, Bcl-x_L_ levels may be upregulated to protect against certain forms of injury. For example, liver cells with decreased Bcl-x_L_ levels demonstrate increased susceptibility to injury [17],[18]; conversely, transgenic overexpression of Bcl-x_L_ protects against liver injury [19]. In this context, it is intriguing that hepatic Bcl-x_L_ expression is upregulated in response to liver injury [20],[21]. Similarly, Bcl-x_L_ levels are upregulated in the esophageal mucosa in response to chronic acid reflux [22]. It is likely that the increased Bcl-x_L_ in these and other instances protects against tissue injury.

The findings outlined above underscore the importance of understanding the mechanisms by which Bcl-x_L_ levels are regulated. We and others have previously shown that two asparagines in human Bcl-x_L_ undergo deamidation to aspartyl or isoaspartyl residues and that the rate of deamidation of these asparagines increases in susceptible tumor cells that are treated with DNA-damaging agents [16],[23],[24]. We now present evidence that asparagine deamidation has been conserved in Bcl-x_L_-like proteins from the simplest extant metazoans through the human form of Bcl-x_L_. This extent of conservation suggests that deamidation has a critical role as a regulatory posttranslational modification of Bcl-x_L_. Indeed, we demonstrate here that the rate of deamidation dynamically modulates the cellular level of Bcl-x_L_ because deamidation is a continuous but regulated process that, like phosphorylation in other proteins, activates a conditional PEST sequence to target Bcl-x_L_ for degradation. Importantly, we show that in susceptible tumor cells, DNA-damaging agents decrease Bcl-x_L_ levels, which increases cellular susceptibility to death signaling, because these agents induce an increase in the rate of deamidation of Bcl-x_L_ and, consequently, an increase in the rate of degradation of Bcl-x_L_. In contrast, however, we have previously shown that at least in some nontransformed cells the increased rate of Bcl-x_L_ deamidation and consequent degradation that would otherwise occur upon treatment with DNA-damaging antineoplastic agents is suppressed by p53-retinoblastoma protein (pRb) signaling; hence, Bcl-x_L_ levels remain static in these cells when they are treated with DNA-damaging antineoplastic agents [16]. Therefore, Bcl-x_L_ deamidation is a regulatable process and certain stimuli can shift the balance of cellular prosurvival and prodeath activity by altering the rate of Bcl-x_L_ deamidation.

## Results

### Bcl-x_L_ Deamidation Is Highly Conserved

Asparagine deamidation is a nonenzymatic posttranslational modification. Although asparagine deamidation occurs spontaneously, its rate can be regulated by changes in the pH, ionic composition, or temperature of the surrounding cellular microenvironment [25]. An asparagine is most susceptible to deamidation when it is immediately followed by a glycine in a conformationally flexible region of a protein because deamidation is initiated when the peptide bond nitrogen of the N+1 amino acid attacks the carbonyl carbon of the asparagine side chain—this is facilitated by the reduced steric hindrance of glycine and flexibility of the surrounding sequence [25].

**Figure 1:**
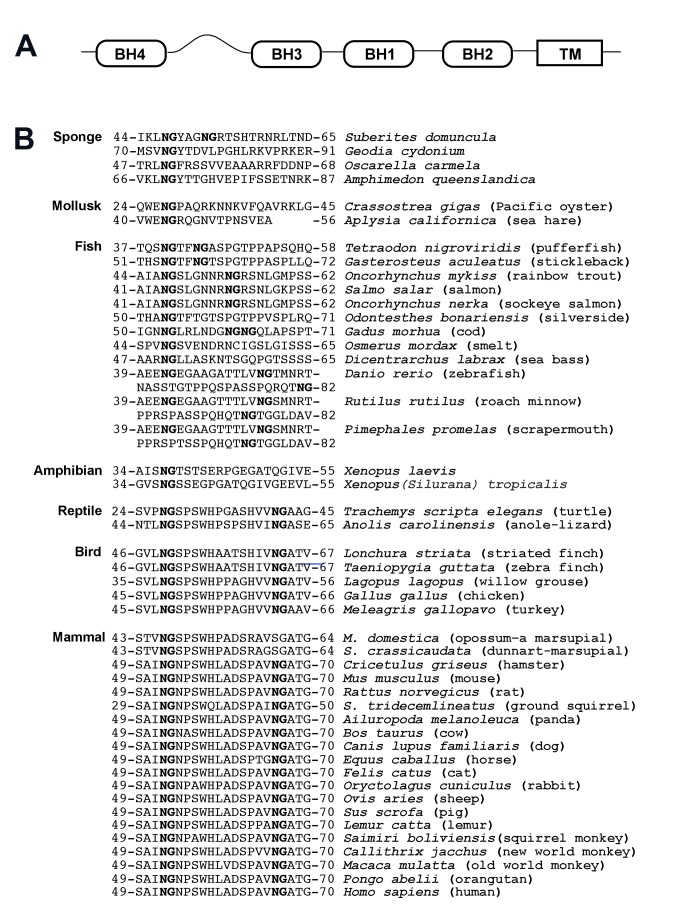
Asparagine-glycine sequences are present in a flexible region between the BH4 and BH3 domains of Bcl-x_L_-like proteins from a wide range of species. (A) Schematic demonstrating the relationship between the four Bcl-2 homology (BH) domains, the transmembrane domain (TM), and the conformationally flexible region (curved line) that lies between the BH4 and BH3 domains of human Bcl-x_L_. (B) A comparison of the sequences between the BH4 and BH3 domains of Bcl-x_L_-like proteins. The sequence surrounding the asparagine-glycine sequence (bolded) in each of these proteins is predicted to be flexible when analyzed by PSIPRED/JPRED[27],[28]. Genbank accession numbers for these proteins are listed in Table S1.

**Figure 2:**
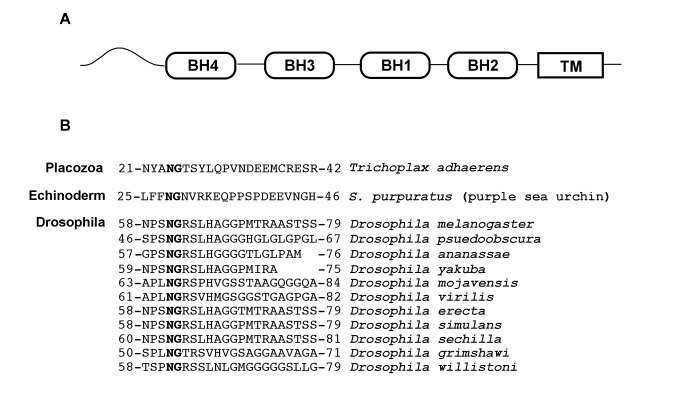
Asparagine-glycine sequences are present in a flexible region that is upstream of the BH4 domain of the Bcl-x_L_-like proteins in several species. (A) Schematic demonstrating the position of a unique region (curved line) upstream of the BH4 domain that is predicted to be flexible when analyzed by PSIPRED/JPRED [27],[28] that is present in the Bcl-x_L_–like proteins of several species. (B) Each of these proteins contains an asparagine-glycine sequence (bolded) in the flexible region. Genbank accession numbers for these proteins are listed in Table S1.

Human Bcl-x_L_ has a large conformationally flexible region between the BH4 and BH3 domains that is referred to as its flexible loop (Figure 1A) [26] and we have previously demonstrated that two asparagines that are immediatedly followed by glycines that lie within the flexible loop undergo deamidation [16]. We now report that Bcl-x_L_-like proteins from sponge through human contain asparagine-glycine sequences within a region that is predicted to be conformationally flexible between the BH4 and BH3 domains (Figure 1B) [27],[28]. The widespread presence of these asparagine-glycine sequences in the flexible region is striking as there is no other obvious sequence similarity within this region across all species (Figure 1B), and it suggests that the presence of an asparagine-glycine sequence per se is a conserved feature of the flexible loop of Bcl-x_L_. Additionally, there are a number of species that express Bcl-x_L_–like proteins that have a long flexible region immediately upstream of the BH4 domain (Figure 2A), and we found that each of these proteins contains an asparagine-glycine sequence in this region (Figure 2B), suggesting that an asparagine-glycine sequence within a flexible region is a conserved feature of Bcl-x_L_.

To objectively assess whether asparagine-glycine sequences are indeed a conserved feature of the Bcl-x_L_ flexible loop, we performed a de novo analysis of an independently assembled group of Bcl-x_L_-like proteins, the Bcl-x_L_ homology group of the Bcl-2 family database[29], using the MEME conserved motif discovery algorithm[30]. MEME is a widely used tool that searches for sequences that are reiterant within an input group of proteins and assigns each an *E*-value, a statistical estimate of the probability that the sequence would occur with an equal or greater frequency than it occurs in the input group of proteins if the amino acids of the proteins were positionally randomized[31]. Sequences with *E*-values of less than 1×1^−2^ likely represent conserved, and therefore functional, motifs[32].

**Figure 3:**
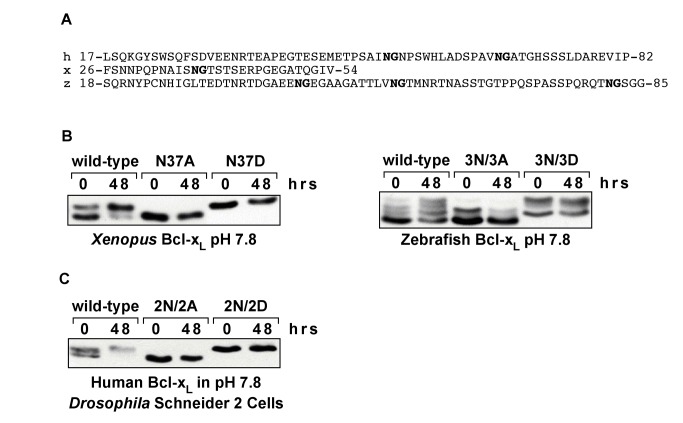
Deamidation is conserved in Bcl-x_L_-like proteins. (A) Sequences from the flexible loops of human (h), *Xenopus laevis* (x), and zebrafish (z) Bcl-x_L_. Asparagine-glycine sequences are bolded. (B) Anti-HSV immunoblots of HSV-tagged wild-type *Xenopus* and zebrafish Bcl-x_L_ and mutant forms of *Xenopus* and zebrafish Bcl-x_L_ in which the asparagines of the asparagine-glycine dipeptides in the flexible loop of each protein are substituted with the indicated amino acids. We have previously demonstrated that the most rapidly migrating form of human Bcl-x_L_ during SDS-PAGE is the native form and the forms that migrate more slowly are deamidated at one or both sites[16]. *Xenopus* Bcl-x_L_ was expressed in SAOS-2 cells and zebrafish Bcl-x_L_ was expressed in C33a cells and lysates of the cells were incubated in a pH 7.8 buffer for the number of hours indicated. Similar results were obtained when *Xenopus* Bcl-x_L_ was expressed in C33a cells and zebrafish Bcl-x_L_ was expressed in SAOS-2 cells. (C) Anti-HA immunoblots of HA-tagged wild-type human Bcl-x_L_, human Bcl-x_L_(N52A/N66A), and human Bcl-x_L_(N52D/N66D). Human Bcl-x_L_ was expressed in *Drosophila* Schneider 2 cells and lysates of the cells were incubated in a pH 7.8 buffer for the number of hours indicated.

In a dataset consisting of the sequences of the entire region between the BH4 and BH3 domains from all of the members of the Bcl-x_L_ homology group in the Bcl-2 database (Figure S1) [29], the asparagine-glycine dipeptide occurs in more sequences and with a greater frequency than any other dipeptide and it is assigned an *E*-value of 2.0×10^−3^ by MEME. Furthermore, almost half (91/187) of the asparagines in the dataset are a component of an asparagine-glycine sequence. These findings strongly suggest that there is selective pressure to maintain the asparagine-glycine sequence in this region. This implies that deamidation is a conserved feature of the Bcl-x_L_ flexible loop because, to our knowledge, the only function asparagine-glycine dipeptides could have in this context is to serve as deamidation sites. We examined this further by determining if deamidation occurs within three flexible loops in which the asparagine-glycine sequences are surrounded by widely disparate sequences: the flexible loops in the human, *Xenopus laevis*, and zebrafish forms of Bcl-x_L_ (Figure 3A).

We have previously demonstrated that two asparagines in the flexible loop of human Bcl-x_L_ undergo deamidation[16]. Deamidation is readily detected in human Bcl-x_L_ because the deamidated forms migrate more slowly than the native form during SDS-PAGE[16]. The more slowly migrating forms do not develop—that is, deamidation does not occur, if the susceptible asparagines are mutated to alanines to block deamidation. Conversely, Bcl-x_L_ in which these asparagines are mutated to aspartates to mimic deamidation migrates at the same rate as the more slowly migrating, deamidated forms of wild-type Bcl-x_L_ [16]. Additionally, deamidation, and therefore, the development of the more slowly migrating forms, can be further induced by incubating Bcl-x_L_ at an alkaline pH in vitro[16].

We assessed the asparagine-glycine sequences in the flexible loops of Bcl-x_L_ from *Xenopus laevis* (*Xenopus* Bcl-x_L_) and zebrafish for deamidation using the approach outlined above. Wild-type *Xenopus* Bcl-x_L_, which contains a single asparagine-glycine sequence in its flexible loop (Figure 3A), forms a doublet when it is expressed in mammalian cells and evaluated by SDS-PAGE (Figure 3B), but only the upper band forms during SDS-PAGE when the cell extract containing the wild-type *Xenopus* Bcl-x_L_ is first incubated at an alkaline pH (Figure 3B); wild-type zebrafish Bcl-x_L_, which contains three asparagine-glycine sequences in its flexible loop (Figure 3A), forms multiple bands (Figure 3B), and there is a relative decrease in the most rapidly migrating band with a concomitant increase in the more slowly migrating bands when it is incubated at an alkaline pH (Figure 3B). Mutation of the asparagines of the asparagine-glycine sequences to alanines to block deamidation in Xenopus Bcl-x_L_ and zebrafish Bcl-x_L_ [*Xenopus* Bcl-x_L_(N37A) and zebrafish Bcl-x_L_(3N/3A), respectively] blocks the formation of the more slowly migrating bands (Figure 3B); mutation of the asparagines to aspartates to mimic deamidation yields forms of Bcl-x_L_ [*Xenopus* Bcl-x_L_(N37D) and zebrafish Bcl-x_L_(3N/3D), respectively] that migrate with the upper bands of their respective wild-type proteins (Figure 3B). Finally, the mutant forms of *Xenopus* and zebrafish Bcl-x_L_ are unaffected when incubated in an alkaline buffer (Figure 3B) (we note that there is a protein band that is most readily visualized in the lanes of the two mutant forms of zebrafish Bcl-x_L_ that migrates at an intermediate rate and appears to be unaffected by incubation in an alkaline buffer—the nature of this band is unknown). These findings demonstrate that the human, *Xenopus laevis*, and zebrafish forms of Bcl-x_L_ all have the potential to undergo deamidation.

That deamidation could occur at asparagine-glycine sequences in flexible loops with such disparate sequences as those in human, *Xenopus laevis*, and zebrafish Bcl-x_L_-like proteins is consistent with the finding that asparagines that are followed by glycines in flexible regions of proteins are exquisitely labile to deamidation [25],[33] and the finding in model peptides that the deamidation rate is determined primarily by the amino acid that immediately follows the asparagine with the amino acids in surrounding positions having little or no effect [33]. When considered in this context, our findings suggest that deamidation could occur at the asparagine-glycine sequences in the flexible loops of Bcl-x_L_-like proteins irrespective of the immediate surrounding sequence. Therefore, our findings suggest that deamidation is a feature of the flexible loop of Bcl-x_L_-like proteins across a wide range of species.

We next wanted to determine if Bcl-x_L_ deamidation occurs in nonmammalian cells. When expressed in *Drosophila* Schneider 2 cells and analyzed by SDS-PAGE, we found that wild-type human Bcl-x_L_ forms a doublet (Figure 3C). The lower band of the doublet migrated with a mutant form of human Bcl-x_L_ in which the deamidation is blocked by replacement of the asparagines with alanines [Bcl-x_L_(N52A/N66A)] [16], while the upper band of the doublet migrated with a mutant human Bcl-x_L_ construct in which the susceptible asparagines are replaced with aspartates to generate a constitutively deamidated form of Bcl-x_L_ [Bcl-x_L_(N52D/N66D)] (Figure 3C) [16]. Additionally, when the *Drosophila* Schneider 2 cell lysates were incubated at an alkaline pH prior to SDS-PAGE, the wild-type Bcl-x_L_ migrated in the position of the upper band of the doublet, while the mutant forms were unaffected (Figure 3C). These findings suggest that the lower band of the doublet found in intact *Drosophila* Schneider 2 cells is the native form of human Bcl-x_L_ and the upper band of the doublet is deamidated Bcl-x_L_. That Bcl-x_L_ deamidation occurs in both insect and human cells strongly suggests that deamidation of Bcl-x_L_–like proteins can occur in a wide range of species.

### Deamidation Targets Human Bcl-x_L_ for Degradation

The rate of Bcl-x_L_ deamidation is increased in response to treatment with DNA-damaging agents, such as cisplatin, etoposide, and γ-radiation, in several types of tumor cells [16]. We and others have found that a form of Bcl-x_L_ in which deamidation is blocked affords tumor cells increased resistance to these agents when compared to the effect of wild-type Bcl-x_L_ [16],[23],[24]. Additionally, Zhao and coworkers found that the suppression of Bcl-x_L_ deamidation by an oncogenic tyrosine kinase contributes to etoposide and γ-radiation resistance in a mouse tumor model [23] and in human myeloproliferative disorders [34], and there is evidence that suppression of Bcl-x_L_ deamidation is a component of hepatocellular carcinogenesis [35]. These findings suggested that deamidation decreases cellular Bcl-x_L_ prosurvival activity.

We originally reported that deamidation decreases the prosurvival activity of Bcl-x_L_ by disrupting its ability to sequester prodeath Bcl-2 family members such as Bim in vivo [16]; however, we subsequently found that our conclusion was based on artifactual results (please see erratum, reference [36]). Surprisingly, though, another group has since published that deamidation does indeed disrupt the ability of Bcl-x_L_ to sequester Bim both in vivo and when in solution in vitro [23],[37]. Their in vitro findings were particularly surprising because (i) the deamidation sites are positioned near the center of the large unstructured region of Bcl-x_L_; (ii) the unstructured region is not necessary for the interaction with Bim or for the antiapoptotic activity of Bcl-x_L_ [38]; (iii) the unstructured region remains unstructured in the deamidated form of Bcl-x_L_ [39]; and (iv) the native and deamidated forms of Bcl-x_L_ “adopt an essentially identical backbone structure” in solution [39]. Therefore, we reexamined the effect of deamidation on the ability of Bcl-x_L_ to bind Bim using several different approaches and controls. We found that deamidation has no effect on the ability of Bcl-x_L_ to bind Bim or Bax (Text S1 and Figure S2). This is consistent with the finding that both the native and deamidated forms of Bcl-x_L_ bind equally to PGAM5, a protein that has been implicated in oxidative stress-induced apoptosis [40]. Furthermore, Bcl-x_L_ encodes several additional presumed prosurvival activities, such as the ability to bind to p53 [41]–[43] and the ability to regulate mitochondrial membrane permeability by forming an ion channel [44]–[46]. It seemed unlikely that deamidation within the unstructured loop could directly inactivate all of these functions. Therefore, we sought the mechanism by which deamidation decreases cellular Bcl-x_L_ prosurvival activity.

**Figure 4:**
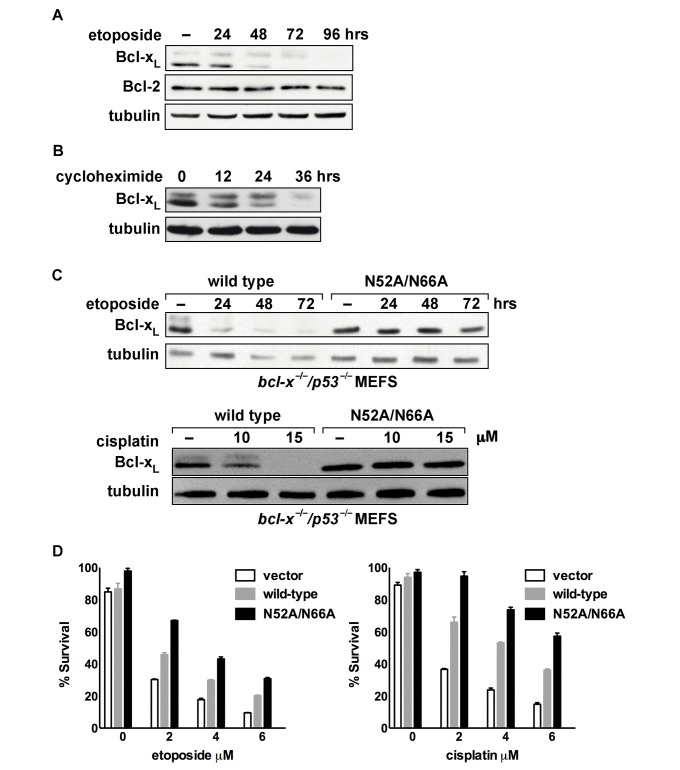
Deamidation targets Bcl-x_L_ for degradation. (A) Immunoblot of endogenous Bcl-x_L_, Bcl-2, and tubulin from SAOS-2 cells that were treated with 10 µM of etoposide for the indicated times. (B) Immunoblot of endogenous Bcl-x_L_ and tubulin in SAOS-2 cells that were treated with 10 µg/ml of cycloheximide for the indicated times. (C) Immunoblot of Bcl-x_L_ and tubulin in *bcl-x^−/−^*/*p53^−/−^* MEFs expressing wild-type Bcl-x_L_ and a form of Bcl-x_L_ in which deamidation is disrupted by substitution of alanines for the two susceptible asparagines, Bcl-x_L_(N52A/N66A). The cells were treated with 5 µM of etoposide for the indicated times or with the indicated concentration of cisplatin. (D) Survival assays of *bcl-x^−/−^/p53^−/−^* MEFs expressing Bcl-x_L_(N52A/N66A) and wild-type Bcl-x_L_. MEFs were treated with etoposide and cisplatin and assessed for apoptosis after 72 h.

**Figure 6:**
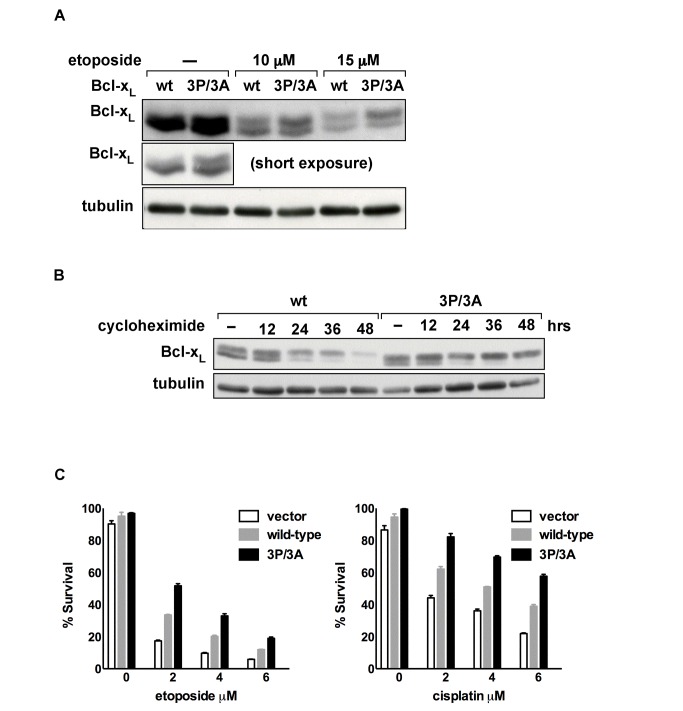
Deamidation activates a conditional PEST sequence to target Bcl-x_L_ for degradation. (A) Immunoblot of Bcl-x_L_ in *bcl-x^−/−^/p53^−/−^* MEFs infected with vectors for wild-type Bcl-x_L_ or a form of Bcl-x_L_ in which the PEST sequence is disrupted by substitution of alanines for three of the PEST sequence prolines, Bcl-x_L_(3P/A), that were treated with etoposide as indicated. Two different exposures of the immunoblot are shown to facilitate the visualization of deamidated forms of Bcl-x_L_. (B) Anti-HA and tubulin immunoblot of 2 µg/ml of cycloheximide-treated SAOS-2 cells expressing HA-tagged versions of wild-type Bcl-x_L_ or Bcl-x_L_(3P/A) for the indicated times. (C) Survival assay of *bcl-x^−/−^/p53^−/−^* MEFs expressing Bcl-x_L_(3P/3A) and wild-type Bcl-x_L_. MEFs were treated with etoposide or cisplatin as indicated and survival was assessed at 48 h.

We noted that the levels of endogenous Bcl-x_L_ decreased as deamidation increased in several of our previous experiments (e.g., figure 2 and figure 6 in reference [16]) and a correlation between deamidation and decreased Bcl-x_L_ levels in maturing erythrocytes was noted by Koury and coworkers [47]. Furthermore, in cells in which apoptosis was induced by oxidative damage, a fragment of Bcl-x_L_, but not full-length Bcl-x_L_, was found to be bound to an enzyme that binds deamidated proteins [48], which suggests that Bcl-x_L_ is rapidly degraded upon deamidation. Therefore, we considered the possibility that deamidation decreases the cellular activities of Bcl-x_L_ by targeting Bcl-x_L_ for degradation. Indeed, we found a clear correlation between the DNA damage-induced increase in Bcl-x_L_ deamidation and a decrease in Bcl-x_L_ levels (Figure 4A).

To begin to determine if it is specifically the deamidated forms that are targeted for degradation, we first blocked synthesis of the native form of Bcl-x_L_ using cycloheximide. We found that the level of the native Bcl-x_L_ decreases first and then, once the native Bcl-x_L_ is depleted, the level of deamidated Bcl-x_L_ decreases (Figure 4B). The simplest explanation for this finding is that the native Bcl-x_L_ is constantly deamidated, even in cells that have not been treated with DNA damaging agents, and the deamidated forms are degraded.

To confirm that the deamidated forms are specifically targeted for degradation, we compared the stability of wild-type Bcl-x_L_ and a form of Bcl-x_L_ in which deamidation is blocked because the susceptible asparagines are mutated to alanines, [Bcl-x_L_(N52A/N66A)] [16]. We have previously shown that the signaling that increases the rate of Bcl-x_L_ deamidation in cells that are treated with DNA damaging agents is suppressed in wild-type mouse embryo fibroblasts (MEFs) and that the suppression is dependent upon the activation of pRb by p53 signaling [16]. Therefore, to determine if deamidation targets Bcl-x_L_ for degradation we reconstituted Bcl-x_L_ expression in *bcl-x^−/−^*/*p53^−/−^* MEFs with either wild-type Bcl-x_L_ or Bcl-x_L_(N52A/N66A). Importantly, we expressed each protein using retroviral infection at a multiplicity of infection of <1 without polybrene treatment or centrifugation so that instead of overexpressing the Bcl-x_L_ constructs at high levels, we approximated the level of Bcl-x_L_ found in wild-type MEFs as closely as possible. After antibiotic selection for the infected cells, we treated the pooled cells with etoposide or cisplatin to induce increased deamidation of Bcl-x_L_. Whereas the level of wild-type Bcl-x_L_ decreased progressively after etoposide or cisplatin treatment, the level of Bcl-x_L_(N52A/N66A), the form of Bcl-x_L_ in which deamidation is blocked, remained relatively constant (Figure 4C). As would be expected, the cells expressing Bcl-x_L_(N52A/N66A) were more resistant to the apoptotic effects of etoposide and cisplatin than were the cells in which the wild-type Bcl-x_L_ was expressed (Figure 4D). These findings strongly suggest that deamidation mediates the inactivation of Bcl-x_L_ prosurvival activity by mediating the degradation of Bcl-x_L_.

**Figure 5:**
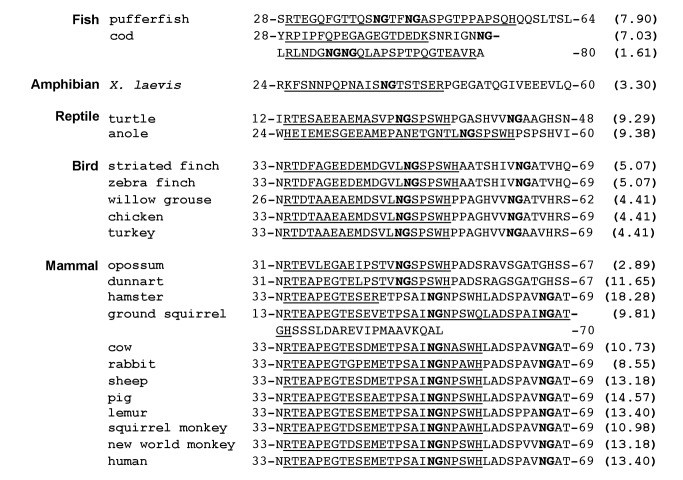
The PEST sequence is conserved among a wide range of nonmammalian and all mammalian forms of Bcl-x_L_. PEST sequences are underlined and asparagine-glycine sequences are bolded. PESTfind scores[78] were calculated for the deamidated form of each protein. Representative mammalian PEST sequences are shown; all of the mammalian forms of Bcl-x_L_ that are listed in Figure 1B contain PEST sequences that are either identical to or near identical to these sequences.

Proteins that are subject to regulatory degradation often contain PEST sequences and the presence of PEST sequences is specific to such proteins—that is, PEST sequences are rarely found in long-lived cellular proteins[49]. PEST sequences are hydrophilic stretches of at least 12 amino acids that are enriched in prolines, glutamates, aspartates, serines, and threonines that are flanked by but do not contain histidines, arginines, or lysines [50]. The PESTfind algorithm identifies potential PEST sequences and assigns them a score that predicts the likelihood that they truly function as a degradation signal sequence [50]. A score above zero denotes a potential PEST sequence[50]; the higher the score, the more likely the sequence functions to target the protein for degradation. Whereas the most well characterized PEST sequence, the PEST sequence in IκBα, has a PESTfind score of 5.90[49], human Bcl-x_L_ contains a PEST sequence with a score of 10.79, which suggested that we would find that the human Bcl-x_L_ PEST sequence truly functions as a proteolytic signaling sequence. It is also notable that (i) the PEST sequence is conserved among all mammalian forms of Bcl-x_L_ (Figure 5); (ii) even though the sequences themselves differ considerably from the mammalian sequence, there are sequences that are identified by the PESTfind algorithm as potential PEST sequences in a similar position in the Bcl-x_L_-like proteins from a wide range of nonmammalian species (Figure 5)—that is, suggesting that there is conservation of a specific function at this position even though the sequence is not conserved; and (iii) PEST sequences only occur infrequently and indeed, there are no other sequences with PESTfind scores greater than zero at any other position within any of the Bcl-x_L_-like proteins listed in Figure 1B. These findings argue strongly for the importance of a functional PEST sequence at this position.

Importantly, a PEST sequence may either constitutively or conditionally target a protein for proteolysis [49]. Therefore, it was intriguing that the PEST sequences either encompass or are in close proximity to the deamidation sites (Figure 5). This was intriguing because phosphorylation within or in proximity to certain conditional PEST sequences increases proteolytic signaling [49] and the products of deamidation, aspartyl residues, can functionally mimic phosphorylated amino acids [51]. Similarly, because deamidation adds an aspartyl residue, it increases the hydrophilicity and, hence, the PESTfind score of the PEST sequence (e.g., the PESTfind score of human Bcl-x_L_ increases from 10.79 to 13.40 upon deamidation), which suggested that deamidation increases the activity of the PEST sequence. Therefore, we assessed the possibility that, like phosphorylation in other proteins, deamidation activates the PEST sequence as a signal for the proteolysis of Bcl-x_L_.

To test this, we generated a human Bcl-x_L_ construct in which the three prolines of the PEST sequence are mutated to alanines [Bcl-x_L_(3P/3A)] to partially disrupt its activity. We found that the level of deamidated Bcl-x_L_(3P/3A) relative to the native form is increased when compared with wild-type Bcl-x_L_ in untreated cells and cells treated with etoposide (Figure 6A) and that this is due to increased stability of the deamidated forms (Figure 6B). Furthermore, the cells expressing Bcl-x_L_(3P/3A) were significantly more resistant to etoposide- and cisplatin-induced apoptosis than those expressing wild-type Bcl-x_L_ (Figure 6C). The simplest explanation for these findings is that the function of Bcl-x_L_ deamidation is to increase the proteolytic targeting activity of the Bcl-x_L_ PEST sequence.

Importantly, in the experiment depicted in Figure 6A and in several of the experiments discussed below, the Bcl-x_L_ constructs are overexpressed and they therefore prevent induction of the later phases of apoptosis. However, even the overexpressed Bcl-x_L_ undergoes an increase in deamidation-regulated degradation upon treatment with DNA-damaging agent agents. This indicates that deamidation-regulated degradation of Bcl-x_L_ is a function of changes that occur in the cell during the premitochondrial phase of apoptosis, the phase in which decreases in Bcl-x_L_ would increase susceptibility to prodeath signaling. This finding and the conservation of the PEST sequence together provide strong evidence of the functional significance of the deamidation-regulated degradation of Bcl-x_L_ as an integral component of the rheostat that regulates cell death.

**Figure 7:**
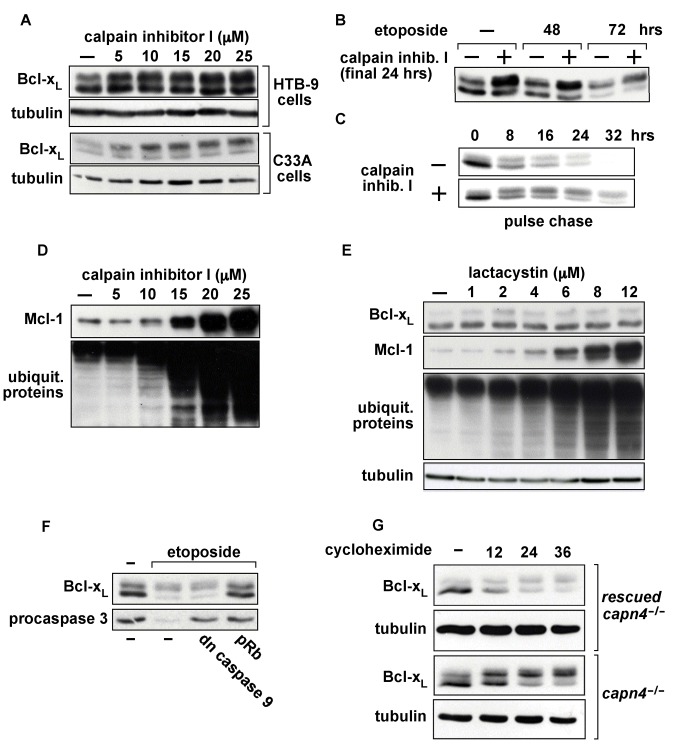
Deamidated Bcl-x_L_ is degraded by calpain. (A) Immunoblots of endogenous Bcl-x_L_ and tubulin in HTB-9 and C33a cells that were treated with calpain inhibitor I for 24 h. (B) Immunoblot of endogenous Bcl-x_L_ from HTB-9 cells that were treated with 10 µM of etoposide for the indicated times and with 10 µM of calpain inhibitor I for the final 24 h of the etoposide treatment. (C) Pulse chase of overexpressed Bcl-x_L_ in Bcl-x_L_–inducible SAOS-2 cells that were treated with 10 µM of calpain inhibitor I as indicated. The contrast of this figure was increased to facilitate visualization of the data. (D) The HTB-9 blot from Figure 7A was reprobed for Mcl-1 and the same lysates that were used for the HTB-9 blot in Figure 7A were probed for total ubiquitinated proteins. (E) Immunoblot of endogenous Bcl-x_L_, Mcl-1, and total ubiquitinated proteins from HTB-9 cells that were treated with the lactacystin for 24 h. Mcl-1 and total ubiquitinated proteins were used as positive controls to evaluate proteasomal activity. (F) Immunoblots of Bcl-x_L_ and procaspase 3 from etoposide-treated SAOS-2 cells in which a dominant negative form of caspase 9 or pRb was expressed. (G) Anti-Bcl-x_L_ immunoblot of *Capn4^−/−^* MEFs that were rescued by expression of *Capn4* and *Capn4^−/−^* MEFs that were treated with cycloheximide for the indicated times. Tubulin was used as a loading control.

Bcl-x_L_ is cleaved by calpain both in vitro and in vivo [52]–[54], which is notable because PEST sequences can target proteins for calpain-mediated degradation [55]–[57]. Therefore, to begin to identify the protease(s) that mediate degradation of deamidated Bcl-x_L_, we treated HTB-9 and C33a cells with calpain inhibitor I and found that it causes primarily an increase of deamidated Bcl-x_L_ in both (Figure 7A). Additionally, the deamidated forms of Bcl-x_L_ are increased by calpain inhibitor I when Bcl-x_L_ deamidation is further induced by etoposide treatment (Figure 7B). Importantly, the increase in the deamidated forms in the cells treated with calpain inhibitor I is due to an increase in stability as assessed by a pulse chase experiment (Figure 7C), demonstrating that calpain inhibitor I increases Bcl-x_L_ levels by blocking its degradation.

Importantly, calpain inhibitor I inhibits several different proteases, not just calpain. In fact, calpain inibitor I also inhibits the proteasome, albeit at a higher concentration than that which is required to inhibit calpain, and PEST sequences can target proteins for proteasomal degradation. We therefore assessed a known proteasomal target, Mcl-1, on the same blot in which we had examined the effect of calpain inhibitor I on Bcl-x_L_ in HTB-9 cells. We also examined total cellular ubiquitinated proteins in the same cell lysates. Whereas 5 µM calpain inhibitor I had caused a near maximal increase in the level of the deamidated forms of Bcl-x_L_ (Figure 7A), Mcl-1 and total ubiquitinated proteins only reached near maximal levels when the cells were treated with 15–20 µM calpain inhibitor I (Figure 7D). We also found that the specific proteasome inhibitor lactacystin had only a relatively small, if any, effect on Bcl-x_L_ compared with its its effect on MCL-1 and total ubiquitinated proteins in HTB-9 cells (Figure 7E). These findings suggest that the proteasomal activity does not have a signficant role in the degradation of the deamidated form of Bcl-x_L_.

Bcl-x_L_ has also been shown to be degraded by caspases [58]. However, we found that stable expression of a dominant negative form of caspase 9 had no effect on Bcl-x_L_ degradation in response to etoposide treatment in SAOS-2 cells even though the dominant negative caspase 9 blocked activation of caspase 3 (Figure 7F) and apoptosis (unpublished data). Expression of the retinoblastoma protein (pRb), which blocks Bcl-x_L_ deamidation [16], was used as a control (Figure 7F). That expression of the dominant negative caspase 9 fails to block degradation of deamidated Bcl-x_L_ is consistent with the finding that overexpressed Bcl-x_L_ is degraded even though its overexpression should block caspase activation. Together these findings demonstrate that caspase activity is not necessary for DNA damage-induced Bcl-x_L_ degradation, at least in certain cell lines.

Finally, to further examine the potential role of calpain in the degradation of deamidated Bcl-x_L_, we examined Bcl-x_L_ in fibroblasts that lack calpain activity [59]. The *Capn4* gene encodes the small subunit of calpain, which is necessary for all calpain activity. When *Capn4^−/−^* MEFs in which calpain activity was rescued by expression of the *Capn4* gene were treated with cycloheximide, Bcl-x_L_ decreased (Figure 7G), as it does in other cells that have calpain activity when they are treated with cycloheximide (e.g., Figures 4B and 6B). However, Bcl-x_L_ accumulated in its deamidated form in *Capn4^−/−^* MEFs when they were treated with cycloheximide. These findings are consistent with a role for calpain in the degradation of deamidated Bcl-x_L_.

### Regulation of Bcl-x_L_ Deamidation by Changes in Cellular pH

It is widely accepted that there is a rapid fall in cytosolic pH of ≈0.3–0.4 units that occurs in apoptosis upon mitochondrial outer membrane permeabilization [60],[61]; however, several groups have reported that cytosolic alkalinization to as high as pH 8.0 occurs early in certain forms of apoptosis, including DNA damage-induced apoptosis [62]–[67]. This is notable because based on structural considerations, Bcl-x_L_ is predicted to be exquisitely susceptible to nonenzymatic deamidation at pH 7.4 [16],[68] and it has been demonstrated that the rate of Bcl-x_L_ deamidation in reticulocyte lysates is increased significantly by increases in pH within the range of pH 7.0 to pH 8.0 [47]. These findings strongly suggested that DNA damage-induced Bcl-x_L_ deamidation is regulated by changes in pH in the cell. Indeed, while this work was in progress, it was confirmed that the DNA damage-induced increase in Bcl-x_L_ deamidation in lymphocytes is induced by the increase in cytosolic pH that occurs in response to DNA damage [37] and we have confirmed that this is also true in the cells of human solid tumors (Text S2 and Figure S3). Notably, the finding that the rate of deamidation is increased by increased pH is further evidence that the DNA damage-induced increase in deamidation of Bcl-x_L_ occurs in the premitochondrial phase of apoptosis, because, as noted above, the onset of the postmitochondrial phase is characterized by a rapid acidification of the cytosol [60],[61], which would be expected to decrease the rate of deamidation of Bcl-x_L_.

**Figure 8:**
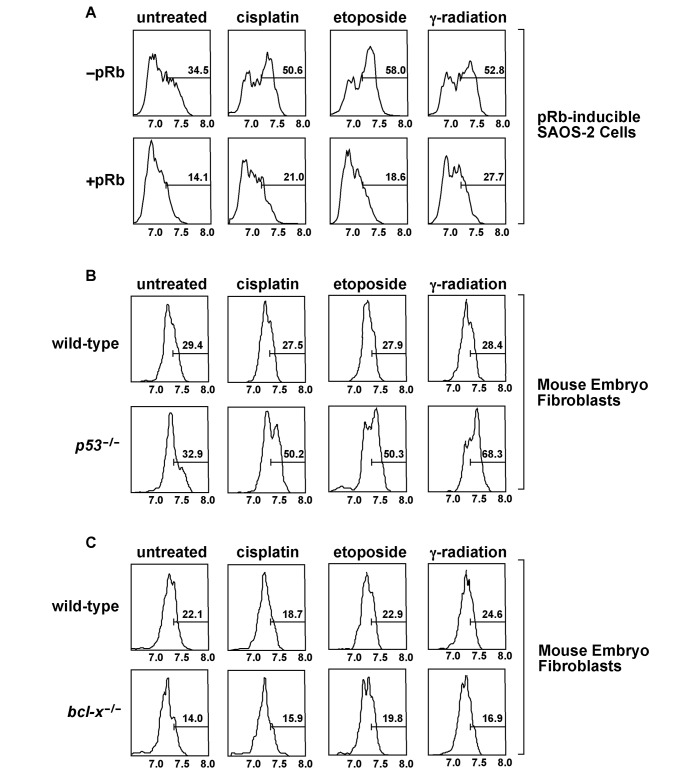
Deamidation-mediated degradation of Bcl-x_L_ is an important function of the DNA damage-induced increase in cellular pH. (A) Rb-inducible SAOS-2 cells were treated with the indicated DNA-damaging agents. Rb expression was induced by treatment with doxycycline prior to DNA-damaging agent treatment. The percent of adherent cells with a pH above an arbitrarily chosen value of approximately pH 7.3 is indicated. (B) Wild-type and *p53^−/−^* MEFs were treated with the indicated DNA-damaging agents and the intracellular pH was measured. (C) Wild-type and *bcl-x^−/−^* MEFs were treated with the indicated DNA-damaging agents and the intracellular pH was measured.

We previously reported that expression of pRb in SAOS-2 osteosarcoma cells blocks both the DNA damage-induced increase in Bcl-x_L_ deamidation and apoptosis [16]. Indeed, we now report that expression of pRb decreases pH in these cells at baseline and after treatment with DNA-damaging agents (Figure 8A). This strongly suggests that Rb blocks an increase in the rate of Bcl-x_L_ deamidation by maintaining the cytoplasm at a relatively low pH after treatment with DNA-damaging agents. This is notable because we found that inhibition of Bcl-x_L_ expression renders SAOS-2 cells that express pRb susceptible to DNA damage-induced apoptosis [16]. Together these findings strongly suggest that the increased rate of deamidation-regulated degradation of Bcl-x_L_ is an important function of the increase in pH that occurs in response to treatment with DNA-damaging agents—that is, alkalinization is necessary to induce an increased rate of deamidation-regulated degradation of Bcl-x_L_, which in turn is necessary for apoptosis to occur, but alkalinization is not necessary if Bcl-x_L_ is absent.

We have also reported that the DNA damage-induced increase in Bcl-x_L_ deamidation is suppressed in wild-type MEFs, but it occurs in *p53^−/−^* MEFs. This is notable because while pRb is activated by DNA damage in wild-type MEFs, it remains inactive after DNA damage occurs in *p53^−/−^* MEFs [16]. Therefore, we hypothesized that the activated pRb in the wild-type MEFs suppresses Bcl-x_L_ deamidation. Consistent with this and our finding that pRb suppresses the alkalinization in SAOS-2 cells, we found that while *p53^−/−^* MEFs are susceptible to the DNA damage-induced alkalinization, wild-type MEFs are not (Figure 8B). Finally, we found that even though *bcl-x^−/−^* MEFs, which have an intact p53-pRb signal transduction pathway, are exquisitely susceptible to apoptosis [16], they do not exhibit a DNA damage-induced increase in cytosolic pH prior to undergoing apoptosis (Figure 8C). This last finding is further evidence that the increased rate of deamidation-regulated degradation of Bcl-x_L_ is an important target of the increase in pH that occurs in response to treatment with DNA-damaging agents in susceptible tumor cells.

## Discussion

Asparagine deamidation was long thought to be a purification artifact; however, in 1968 Flatmark provided the first demonstration that a protein undergoes deamidation within the cell [69]. It is now well accepted that many proteins undergo deamidation within the cell, but deamidation is still viewed nearly universally as a form of protein damage or aging that is detrimental to the organism. This is because deamidation has been thought by most to be an unregulated, spontaneous process that disrupts protein function through the nonspecific disruption of protein structure. Furthermore, whereas deamidation has been implicated in the dysfunction underlying several pathologic processes, such as Alzheimer's disease [70] and cataract formation [71], there has been only limited evidence that it could serve a beneficial role [72].

We have now demonstrated that Bcl-x_L_ deamidation is a process that activates a conditional PEST sequence. The degree of organization underlying both the regulation and functional consequence of Bcl-x_L_ deamidation together with the fact that it is conserved across a wide range of species clearly suggests that deamidation can play a beneficial regulatory role. It is possible that the deamidation that occurs in Alzheimer's disease, cataract formation, and other pathologic processes represents a dysregulated state of a process that normally has an important cellular function. This would be analogous to the contribution of the dysregulation of the phosphorylation of certain proteins to tumorigenesis [73]. Indeed, there is evidence that the dysregulation of Bcl-x_L_ deamidation contributes to the development of hepatocellular carcinoma [35] and myeloproliferative disorders [34]. Notably, in addition to pH, the rate of deamidation is affected by the buffer ion, tonicity, and temperature [74]. A change in any of these that results in a decrease in the rate of Bcl-x_L_ deamidation would have the potential to increase tumor cell viability and inhibit the tumor cell response to treatment, worsening patient outcome.

Additionally, we have shown that even modest changes in Bcl-x_L_ levels can alter the extent of tissue damage in response to certain types of injury [17]. The finding that mutation of the PEST sequence or treatment with calpain inhibitor I in otherwise untreated cells results in a relative increase of the level of deamidated Bcl-x_L_ demonstrates that Bcl-x_L_ levels are continuously modulated by deamidation, even in normally growing cells. Therefore, any change in factors that affects the rate of deamidation could alter the extent of tissue damage in response to certain types of injury.

Finally we note that asparagine deamidation is an extraordinarily simple posttranslational modification in that it only requires a water molecule to proceed. Its simplicity suggests that it was an early form of posttranslational modification. In this context, it is notable that asparagine is the evolutionary offspring of aspartate and it is thought that asparagine “captured” what were originally two aspartate codons to serve as its codons [75]. Thus we speculate that asparagines replaced certain aspartates as proteins evolved so that a residue with an inducible negative charge, asparagine, could replace a residue with a fixed negative charge, aspartate. This substitution would have afforded a greater degree of control of protein function. Indeed, it may have been the selective advantage of the potential to switch from a neutral residue to a charged residue that initially drove the stable incorporation of asparagine into proteins.

## Materials and Methods

### MEME Analysis

The protein sequences listed in the Bcl-x_L_ (BCL2L1) homology group of the Bcl-2 database(Figure S1) [29] that contain both a BH4 and a BH3 domain were identified using the online Batch Search tool of the Conserved Domain Database[76]. The intervening sequences between the BH4 and BH3 domains in these proteins were compiled to form the dataset that was submitted to the MEME server for analysis. Importantly, there are species in the database that express more than one protein in which the sequence between the BH4 and BH3 domains are identical; such proteins are typically the result of alternative splicing. In such instances, the sequence was only included once in the analysis.

### Cell Culture, Plasmids, and Retroviral Constructs

SAOS-2 cells (ATCC HTB-85), C33a cells (ATCC HTB-31), and MEFs were maintained in DMEM with 10% FBS. HTB-9 cells (ATCC HTB-9) were maintained in RPMI-1640 with 10% FBS. *Drosophila* Schneider 2 cells were maintained in Shields and Sang M3 (Sigma) with 10% FBS. *bcl-x^−/−^*, *p53^−/−^*, and *bcl-x^−/−^*/*p53^−/−^* MEFs and Rb-inducible SAOS-2 cells were described previously [16]. Capn4*^−/−^* MEFs were described previously [59]. Bcl-x_L_–inducible SAOS-2 cells were generated using the T-Rex system (Invitrogen). *Xenopus* and zebrafish Bcl-x_L_ cDNAs were amplified by RT-PCR using primers 5′-ATATATCCATGGCAGAGGGCAGCAGTAGAGATCTGGTGG-3′ and 5′-TATATACAGCTGTCGGCGCCTCATGTAGCAGACC-3′ with *Xenopus* mRNA and 5′-ATATATCCTGGCATCTTACTATAACCGAGAACTGGTGG-3′ and 5′-TATATACAGCTGCAGGCGTTTCTGTGCAATGAGTCCCCC-3′ with zebrafish mRNA (the underlined sequences in the primers were used for cloning purposes). The products were cloned between the *Nco* I site and *Pvu* II site in the plasmid pTriEx-1.1 (Novagen). The sequences of the inserts were confirmed as identical to the *Xenopus* Bcl-x_L_ sequence (Genbank accession no. NP001082147) and the zebrafish Bcl-x_L_ sequence (Genbank accession no. NP571882) listed in the NCBI databases. All mutations were made using the QuikChange Kit (Stratagene). *Xenopus* Bcl-x_L_ codon 37 was changed from AAT to GCT to generate *Xenopus* Bcl-x_L_(N37A) and codon 37 was changed from AAT to GAT to generate *Xenopus* Bcl-x_L_(N37D). Zebrafish Bcl-x_L_ codon 42 was changed from AAT to GCT, codon 54 from AAT to GCT, and codon 81 from AAT to GCT to generate zebrafish Bcl-x_L_(3N/3A), and codon 42 was changed from AAT to GAT, codon 54 from AAT to GAT, and codon 81 from AAT to GAT to generate zebrafish Bcl-x_L_(3N/3D). For expression of the wild-type and mutant forms of human Bcl-x_L_ in *Drosophila* Schnieder 2 cells pCMA-Bcl-x_L_, pCMA-Bcl-x_L_(N52A/N66A), and pCMA-Bcl-x_L_(N52D/N66D) were constructed by ligation of PstI Bcl-x_L_ encoding fragments from pSFFV-Bcl-x_L_, pSFFV-Bcl-x_L_(N52A/N66A), and pSFFV-Bcl-x_L_(N52D/N66D) [16] into the PstI site of pCMA [77]. Retroviral vectors for expression of wild-type and mutant forms of human Bcl-x_L_ were generated as follows. The retroviral construct pBABE-blast and pBABE-blast-HA were generated by ClaI/HindIII digest of pBABE-puro and pBABE-puro-HA (removes the puromycin resistance gene) and blunt-end ligation of the blasticidin resistance gene with its promoter from pcDNA/TR (Invitrogen) into these sites. pBABE-blast-Bcl-x_L_, pBABE-blast-Bcl-x_L_(N52A/N66A), and pBABE-blast-HA-Bcl-x_L_ were constructed by ligation of the EcoRI Bcl-x_L_ encoding fragments from pSFFV-Bcl-x_L_ and pSFFV-Bcl-x_L_(N52A/N66A) [16] into the EcoRI site of pBABE-blast and pBABE-blast-HA. In pBABE-blast-Bcl-x_L_ and pBABE-blast-HA-Bcl-x_L_, Bcl-x_L_ codon 38 was changed from CCA to GCA, codon 48 from CCC to GCC, and codon 55 from CCA to GCA to generate pBABE-blast-Bcl-x_L_(3P/3A) and pBABE-blast-HA-Bcl-x_L_(3P/3A). pCDNA3-Flag-dominant negative caspase 9 was described previously [16].

### Infection and Transfection

Retroviral particles were produced by transient transfection of Phoenix E cells with either pBABE-blast-Bcl-x_L_ or pBABE-blast-Bcl-x_L_(N52A/N66A). The pBABE-blast-Bcl-x_L_ and pBABE-blast-Bcl-x_L_(N52A/N66A) supernatants were collected from the Phoenix E cell cultures and diluted 1∶5 in fresh media. The diluted retrovirus was added to the medium of the MEFs without polybrene or centrifugation. Twenty-four hours later, blasticidin (1.0 µg/ml) was added to the media. After selection, 1×10^5^ cells were plated on 60 mm dishes and treated 24 h later with 5 µM of etoposide. Standard retroviral techniques were used for assessment of the PEST sequence in MEFs. SAOS-2, HTB-9, and C33a cells were transfected using the calcium phosphate method. *Drosophila* Schneider 2 cells were transfected using nucleofector (Amaxa). Survival was quantified by flow cytometry using the Live/Dead kit (Molecular Probes) or by microplate reader at 450 nm using the Cell Counting kit-8 (Dojindo Molecular Technologies).

### Immunoblotting and Immunoprecipitation

The following antibodies were used: anti-Bcl-x_L_ (610211) and anti-Bcl-2 (610538) from Transduction Laboratories; anti-Bcl-x_L_ (2764) from Cell Signaling; anti-tubulin (sc-9104), anti-actin (sc-1616), anti-Mcl-1 (sc-819), and anti-Ubiquitin (sc-8017) from Santa Cruz Biotechnology; anti-HSV-Tag (69171) from Novagen; and anti-HA (1867423) from Roche. Immunoblotting and immunoprecipitation were performed as previously described [16]. For immunoprecipitation, lysis buffer (50 mM HEPES (pH 7.0), 250 mM NaCl, 1 mM EDTA, 0.2% NP-40, and Complete Protease Inhibitor (Roche) was used.

### Pulse Chase

Bcl-x_L_–inducible SAOS-2 cells were induced by doxycycline treatment and pulsed with ^35^S-methionine for 4 h. Cells treated with calpain inhibitor I as indicated and chased for the specified times. The cell lysates were prepared and immunoprecipitated for Bcl-x_L_ as described previously [16] and then analyzed by SDS-PAGE and autoradiography.

### Intracellular pH Measurements

Cells were grown in a HEPES-buffered medium instead of the standard HCO_3_^−^/CO_2_ buffer system to avoid the rapid shifts in pH that occur when cells in the HCO_3_^−^/CO_2_ buffer system are removed from the 5% CO_2_ atmosphere of an incubator. Sixty hours after DNA-damaging agent treatment, cells were washed with PBS. The studies were purposefully biased towards the assessment of cells in the earlier stages of apoptosis by measuring the pH of only those cells that remained adherent to the tissue culture dish. These cells were loaded with 5 µM of SNARF-1 for 10 min and then washed with PBS just prior to assessment by flow cytometry. The SNARF-1 was excited at 488 nm and emissions were read at 585 nm (pH-dependent) and 640 nm (pH-independent). The pH-independent emission allows for the normalization of SNARF-1 loading differences between cells. The ratio of the emissions was calculated and the pH was read from a calibration curve. An in situ calibration curve was generated as follows: Cells are loaded with SNARF-1 as above. HEPES was used to make buffers at 0.5 pH unit intervals ranging from pH 6.5–8.5. These contained the ionophore nigericin 13 µM and K^+^ 140 mM to render the cells permeable to the buffers. Cells were equilibrated in the buffers for 20 min. The cells were then analyzed by flow cytometry, and a calibration curve of the pH versus the ratio of the pH-dependent and pH-independent emissions was plotted.

## Supporting Information

Figure S1 Dataset used for MEME analysis. The sequences of the region between the BH4 and BH3 domains of the members of the Bcl-x_L_ homology group in the Bcl-2 database[29] that were used for the MEME analysis. Bcl-2 database IDs are listed.
(PDF)

Figure S2 Deamidation has no effect on the interaction of Bcl-x_L_ with Bim or Bax. (A) Immunoblot for endogenous Bcl-x_L_ in whole cell lysates and either IgG (control) or anti-Bim immunoprecipitates from untreated and 10 µM of etoposide-treated C33a cells. We have previously demonstrated that the most rapidly migrating form of human Bcl-x_L_ during SDS-PAGE is the native form and the forms that migrate more slowly are deamidated at one or both sites [16]. (B) Bim expression was induced by doxycycline treatment of a Bim-inducible SAOS-2 cell line in which Bcl-x_L_ is constitutively overexpressed and an immunoblot was performed for the indicated proteins in whole cell lysates and either IgG or anti-Bim immunoprecipitates. Two different exposures of the immunoblot for the co-immunopreciptated proteins are shown to facilitate the visualization of all of the forms of Bcl-x_L_ that are co-immunopreciptated by different concentrations of Bim. That Bcl-x_L_ levels appear to increase after Bim expression is induced is most likely because the cells that express the highest levels of Bcl-x_L_ have a survival advantage once Bim is expressed. (C) The experiment outlined in (B) was repeated using cells that were treated with 10 µM of etoposide to induce further deamidation of Bcl-x_L_. Etoposide treatment depresses the inducibility of Bim in these cells. (D) Immunoblot analysis of Bcl-x_L_ in whole cell lysates (left) and either anti-Bim or anti-HA immunoprecipitates (right) from C33a cells in which HA-tagged Bcl-x_L_ and untagged Bcl-x_L_ were expressed as indicated. The cells were treated with 10 µM of etoposide for 48 h. Both immunoprecipitations were performed using the same cell lysate. (E) GFP-Bax was expressed in a SAOS2 cell line in which Bcl-x_L_ is constitutively overexpressed and an immunoblot was performed for the indicated proteins using whole cell lysates and either IgG or anti-GFP immunoprecipitates. Two different exposures of the immunoblot for the co-immunopreciptated proteins are shown to facilitate the visualization of all of the forms of Bcl-x_L_ that are co-immunopreciptated by different concentrations of the anti-GFP antibody.
(TIF)

Figure S3 Bcl-x_L_ deamidation is regulated by cytosolic pH. (A) Immunoblot of Bcl-x_L_ in SAOS-2 cell lysates that were incubated at pH 7.2, pH 7.4, or pH 7.6 for the indicated times. (B) Immunoblot of Bcl-x_L_ from intact C33a, HTB-9, and SAOS-2 cells that were incubated in tissue culture medium at pH 6.5 or pH 7.3 and treated with 10 µM of monensin as indicated. (C) Immunoblot of purified bacterially synthesized wild-type Bcl-x_L_ and Bcl-x_L_(N52A/N66A) that was incubated at pH 7.2, pH 7.4, or pH 7.6 for the indicated times. (D) SAOS-2 cells were treated with the indicated DNA-damaging agents. The percent of adherent cells with a pH above an arbitrarily chosen value of approximately pH 7.3 is indicated.
(TIF)

Table S1 Genbank accession numbers for Bcl-x_L_-like proteins. Genbank accession numbers for the proteins in Figures 1B and 2B are listed.
(DOCX)

Text S1 Deamidation has no effect on the interaction of Bcl-x_L_ with Bim or Bax.
(DOCX)

Text S2 DNA damage-induced Bcl-x_L_ deamidation is regulated by changes in pH in the cell.
(DOCX)

## Abbreviations

MEF: mouse embryo fibroblast
pRb: retinoblastoma protein
