# Supplementary material for: Control of Cellular Bcl-xL Levels by Deamidation-Regulated Degradation
Source: PLoS Biol. 2013 Jun 25;11(6):e1001588. doi: 10.1371/journal.pbio.1001588 (PMC3692414; doi:10.1371/journal.pbio.1001588)
Supplement: Figure S1 — Dataset used for MEME analysis. The sequences of the region between the BH4 and BH3 domains of the members of the Bcl-xL homology group in the Bcl-2 database[29] that were used for the MEME analysis. Bcl-2 database IDs are listed. (PDF) [file pbio.1001588.s001.pdf]

Fish

112 *Danio rerio*
28-GLTEDTNRTDGAEE**NG**EGAAGATTLV**NG**TMNRTNASSTGTPPQSPASSPQRQT**NG**SGGLDAVKEA-92
113 *Gasterosteus aculeatus*
24-TSLLRPEDAGGRTEGDKANSASVPGRGSSAGQPGMSSPPPPPSGDTEAVKAA-75
520 *Gasterosteus aculeatus*
27-GLSEPPNRTGGGVEAGAAGGQRGATHS**NG**TF**NG**TSPGTPPASPLLQQRSPSTASLDAVKEA-87
471 *Leucoraja erinacea*
16-LQQRGHSWSRLCEQDGAAERG-36
515 *Ochotona princeps*
28-SDVDDTRTEAPEGIEPEMETPSTL**NG**TPAWHPADSPAL**NG**ATGPSSSLDAREVIPMSAVKQA-89
525 *Oncorhynchus mykiss*
25-CQLVLEGASGRTEGDEAIA**NG**SLGNNR**NG**RSNLGMPSSAQGGIEAVKAA-73
524 *Oncorhynchus nerka*
25-CQLVLEGASGRTEGDEAIA**NG**SLGNNR**NG**RSNLGMPSSAQGGIEAVKAA-73
114 *Oryzias latipes*
50-SSEDGRLRRSCPCCASMDAIKST-72
527 *Osmerus mordax*
27-ELTSALPERPGGGEGGASPHA**NG**AP**NG**TSPGTPPPAQPPSSPRRRPSLGAGLDEVKEA-84
526 *Osmerus mordax*
24-ISQLGLEDASERTNVDKTNVSPV**NG**SVENDRNCIGSLGISSSPQGGIEAVKAA-76
523 *Pimephales promelas*
28-GLTEDTNRTDGAEE**NG**EGAAGTTTLV**NG**SMNRTPPRSPTSSPQHQT**NG**TGGLDAVKEA-85
528 *Poecilia reticulata*
63-TSPGSPRRQQAASASTMDAVKVT-85
521 *Rutilus rutilus*
28-GLTEDTNRTDGAEE**NG**EGAAGTTTLV**NG**SMNRTPPRSPASSPQHQT**NG**TGGLDAVKEA-85
522 *Salmo salar*
25-CQLVLEGASGRTEGDEAIA**NG**SLGNNR**NG**RSNLGKPSSPQGGIEAVKAA-73
467 *Squalus acanthias*
25-RLCEQDGAAGLGPQPDEGAADWSGRPEAERACQA-58
268 *Takifugu rubripes*
24-TSLLRPEDTDGRTEGEKRSPGAS**NG**LLVRSRNRGGASPSAGAGIEAVNAA-73
268 *Takifugu rubripes*
24-TSLLRPEDTDGRNRGGASPSAGAGIEAVNAA-54
274 *Tetraodon nigroviridis*
24-SSLLRPQEPDGWTEGDKKSPAAV**NG**LLIRNRSRSGASASTGAGVEAVHAA-73

Amphibian

518 *Xenopus laevis*
27-SNNPQPNAIS**NG**TSTSERPGEGATQGIVEEEVLQA-61
111 *Xenopus tropicalis*
27-SSNSQPKGVS**NG**SSEGPGATQGIVGEEVLQA-57

Reptile

519 *Anolis carolinensis*
25-HEIEMESGEEAMEPANETGNTL**NG**SPSWHPSPSHVI**NG**ASEHPELLEEEEEENPRVDVSQT-85

Bird

517 *Anas platyrhynchos*
27-LEEEDENRTELASEAAAVL**NG**SPSWHPPAGQVV**NG**AAVHRSSLEVHELVQSAAVRQA-83
250 *Gallus gallus*
27-LEEEDENRTDTAAEAEMDSVL**NG**SPSWHPPAGHVV**NG**ATVHRSSLEVHEIVRASDVRQA-85
769 *Lonchura striata*
27-LEEEDENRTDFAGEEDEMDGVL**NG**SPSWHAATSHIV**NG**ATVHQSSLEVHEIRRAADVRQA-86
516 *Taeniopygia guttata*
27-LEEEDENRTDFAGEEDEMDGVL**NG**SPSWHAATSHIV**NG**ATVHQNSLEVHEIRRAADVRQA-86

Mammal

100 *Bos taurus*
28-SDVEENRTEAPEGTESDMETPSAI**NG**NASWHLADSPAV**NG**ATGHSRSSDAREVIPMAAVKQA-89
313 *Callithrix jacchus*
29-SDVEENRTEAPEGTDSEMETPSAI**NG**NPSWHLADSPVV**NG**ATGHSSSLDAREVIPMAAVKQA-90
103 *Canis familiaris*
28-SDVEENRTEAPEGTESEMETPSAI**NG**NPSWHLADSPAV**NG**ATGHSSSLDAREVIPMAAVKQA-89
514 *Capra hircus*
15-GATGHSRSLDAREVIPMAAVKQA-37
320 *Choloepus hoffmanni*
28-SDVEENRTEASEGADSEMETPSAI**NG**NPSWHLADSPAV**NG**ATGHSSSLDAREVIPMAAVKQA-89
249 *Dasypus novemcinctus*
28-SDDVEENRTEASEGTESEMETPSAI**NG**NPSWHLADSPAV**NG**ATGHSSSLDAREVIPMAAVKQA-90
716 *Equus caballus*
28-SDVEENRTEAPEGTESEMETPSAI**NG**NPSWHLADSPTG**NG**ATGHSSSLDAREVIPMAAVKQA-89
105 *Erinaceus europaeus*
28-SDVEENRTEASEGTESEMETPSAI**NG**NPSWHLADSPAL**NG**ATGHSSSLDAREVIPMAAVKQA-89
244 *Felis catus*
28-SDVEENRTEAPEGTESEMETPSAI**NG**NPSWHLADSPAV**NG**ATGHSSSLDAREVIPMAAVKQA-89
95 *Homo sapiens*
28-SDVEENRTEAPEGTESEMETPSAI**NG**NPSWHLADSPAV**NG**ATGHSSSLDAREVIPMAAVKQA-89
101 *Loxodonta africana*
28-SDVEENRTGASEGTESEMEIPSAI**NG**NPSRHLADSPAV**NG**ATGHSSSLDAREVIPMAAVKQA-89
661 *Macaca fascicularis*
28-SDVEENRTEAPEGTESEMETPSAI**NG**NPSWHLVDSPAV**NG**ATGHSSSLDAREVIPMAAVKQA-89
99 *Macaca mulatta*
28-SDVEENRTEAPEGTESEMETPSAI**NG**NPSWHLVDSPAV**NG**ATGHSSSLDAREVIPMAAVKQA-89
314 *Microcebus murinus*
28-IDAEENRTEAPEGTESEMETPSAI**NG**NPSWHLADSPPV**NG**ATGHSSSLDAREVIPMAAVKQA-89
104 *Mus musculus*
28-SDVEENRTEAPEETEAERETPSAI**NG**NPSWHLADSPAV**NG**ATGHSSSLDAREVIPMAAVKQA-89
241 *Myotis lucifugus*
28-SDVEENRTEAPEGTESEVETPSAI**NG**NPSWHLVDSPAV**NG**ATGHSSSLDAREVIPMAAVKQA-89
110 *Oryctolagus cuniculus*
28-SDVEENRTEAPEGTGPEMETPSAI**NG**NPAWHPADSPAV**NG**ATGHSSSLDAREVIPMTAVKQA-89
213 *Otolemur garnettii*
28-SDVEENRTEAPEGNESELETPSAI**NG**NPSWHLADSPTV**NG**ATGHSSSLDAREVIPMAAVKQA-89
770 *Ovis aries*
28-SDVEENRTEAPEGTESDMETPSAI**NG**NPSWHLADSPAV**NG**ATGHSRSLDAREVIPMAAVKQA-89

157 *Pan troglodytes*
28-SDVEENRTEAPEGTESEMETPSAI**NG**NPSWHLADSPAV**NG**ATGHSSSLDAREVIPMAAVKQA-89
898 *Pongo pygmaeus*
28-SDVEENRTEAPEGTESEMETPSAI**NG**NPSWHLADSPAV**NG**ATGHSSSLDAREVIPMAAVKQA-89
374 *Pteropus vampyrus*
28-SDVEENRTEAPEGTESEMEPPSAV**NG**NPSWHLADSPAV**NG**ATGHSSSLDAREVIPMAAVKQA-89
900 *Rattus norvegicus*
28-SDVEENRTEAPEETEPERETPSAI**NG**NPSWHLADSPAV**NG**ATGHSSSLDAREVIPMAAVKQA-89
592 *Sminthopsis crassicaudata*
28-EDENRTEAPEGTELPSTV**NG**SPSWHPADSRAGSGATGHSSSLDATHETIPVAAVKQA-84
789 *Sorex araneus*
28-SDVEENRTEAPEGTESEMETPSAI**NG**NPSWHLADSPAL**NG**ATGHSSSSLDAREVIPLAAVKQA-90
170 *Spermophilus tridecemlineatus*
28-SDVEENRTEAPEGTESEVETPSAI**NG**NPSWHLADSPAI**NG**ATGHSSSLDAREVIPMAAVKQA-89
959 *Sus scrofa*
28-TDVEENRTEAPEGTESEAETPSAI**NG**NPSWHLADSPAV**NG**ATGHSSSLDAREVIPMAAVKQA-89
378 *Trichosurus vulpecula*
28-EDETRTEAPEGTEIPSTV**NG**SPSWHPADSRAVSGATGHSSSLDAHETIPVAAVKQA-83
787 *Tupaia belangeri*
28-SDVEENRTEASEGAESELETPSAI**NG**NPSWHLADSPAV**NG**ATGHSSSLDAREVIPMAAVKQA-89
375 *Tursiops truncatus*
28-SDVDENRTEAPEGTESDMETPSAI**NG**NPSWHLADSPAVNVATGHSSSLDAREVIPMAAVKQA-89
