# Supplementary figures and images for: Control of Cellular Bcl-xL Levels by Deamidation-Regulated Degradation
Source: PLoS Biol. 2013 Jun 25;11(6):e1001588. doi: 10.1371/journal.pbio.1001588 (PMC3692414; doi:10.1371/journal.pbio.1001588)

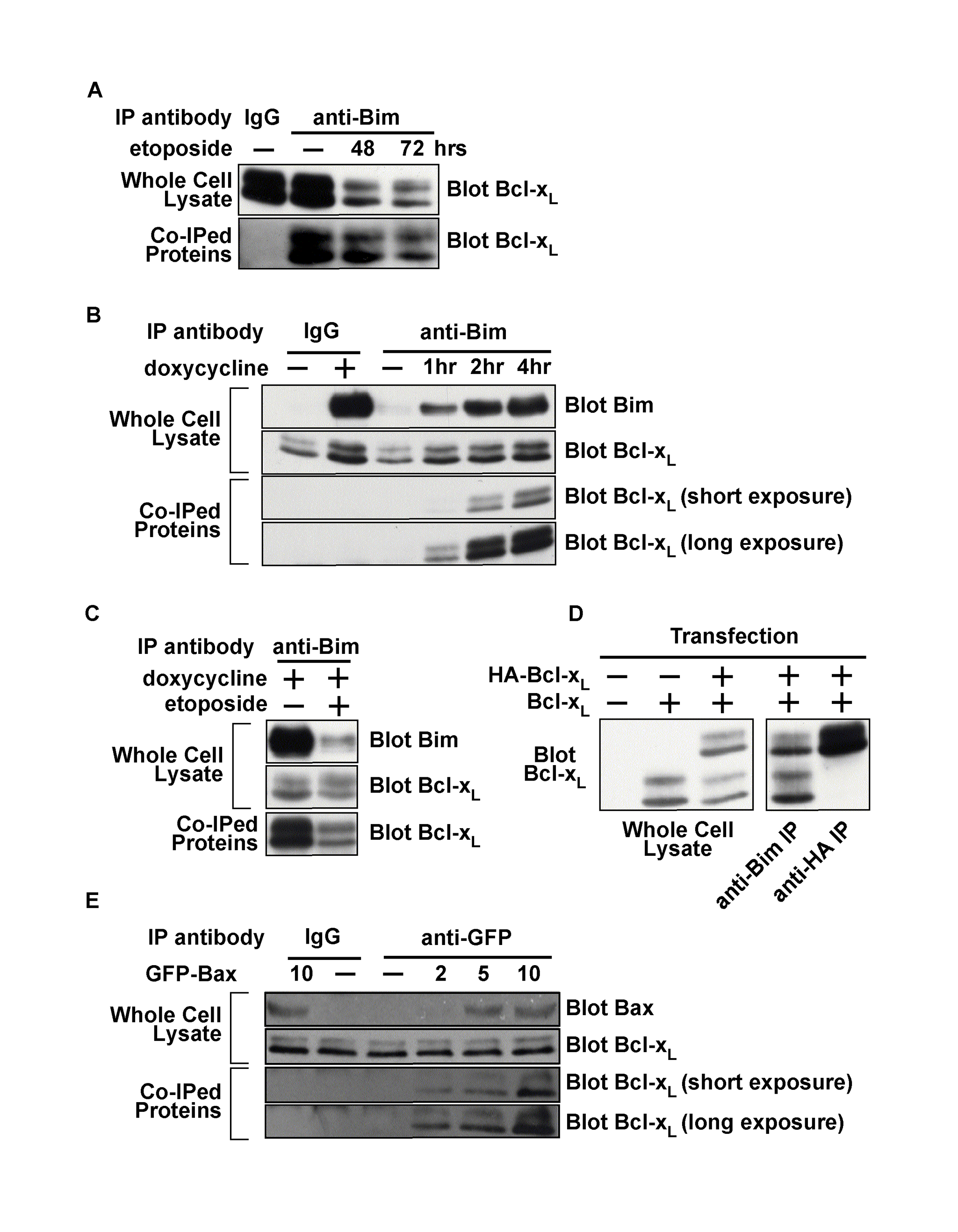

Supplement: Figure S2 — Deamidation has no effect on the interaction of Bcl-xL with Bim or Bax. (A) Immunoblot for endogenous Bcl-xL in whole cell lysates and either IgG (control) or anti-Bim immunoprecipitates from untreated and 10 µM of etoposide-treated C33a cells. We have previously demonstrated that the most rapidly migrating form of human Bcl-xL during SDS-PAGE is the native form and the forms that migrate more slowly are deamidated at one or both sites [16]. (B) Bim expression was induced by doxycycline treatment of a Bim-inducible SAOS-2 cell line in which Bcl-xL is constitutively overexpressed and an immunoblot was performed for the indicated proteins in whole cell lysates and either IgG or anti-Bim immunoprecipitates. Two different exposures of the immunoblot for the co-immunopreciptated proteins are shown to facilitate the visualization of all of the forms of Bcl-xL that are co-immunopreciptated by different concentrations of Bim. That Bcl-xL levels appear to increase after Bim expression is induced is most likely because the cells that express the highest levels of Bcl-xL have a survival advantage once Bim is expressed. (C) The experiment outlined in (B) was repeated using cells that were treated with 10 µM of etoposide to induce further deamidation of Bcl-xL. Etoposide treatment depresses the inducibility of Bim in these cells. (D) Immunoblot analysis of Bcl-xL in whole cell lysates (left) and either anti-Bim or anti-HA immunoprecipitates (right) from C33a cells in which HA-tagged Bcl-xL and untagged Bcl-xL were expressed as indicated. The cells were treated with 10 µM of etoposide for 48 h. Both immunoprecipitations were performed using the same cell lysate. (E) GFP-Bax was expressed in a SAOS2 cell line in which Bcl-xL is constitutively overexpressed and an immunoblot was performed for the indicated proteins using whole cell lysates and either IgG or anti-GFP immunoprecipitates. Two different exposures of the immunoblot for the co-immunopreciptated proteins are shown to faci [file pbio.1001588.s002.tif]

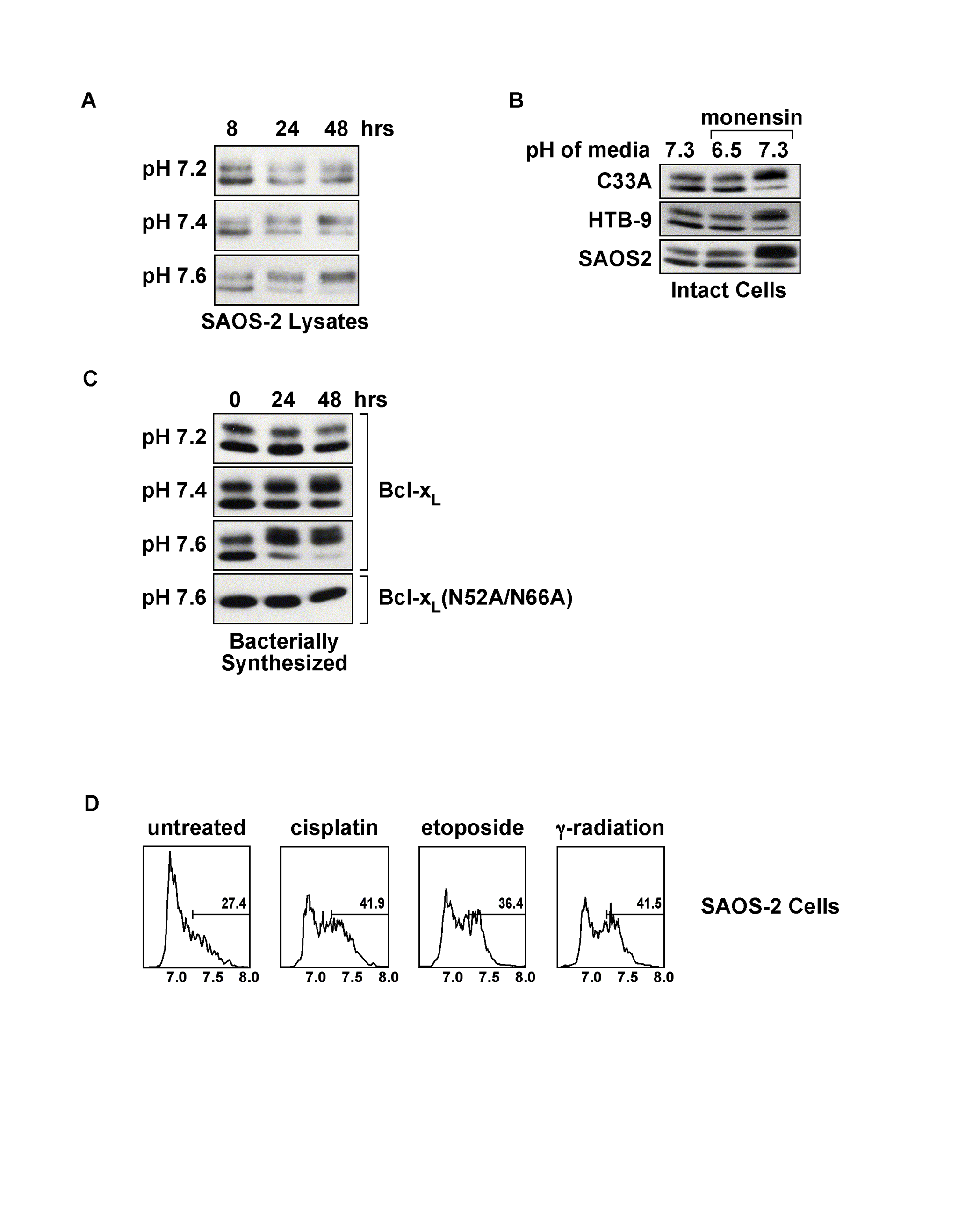

Supplement: Figure S3 — Bcl-xL deamidation is regulated by cytosolic pH. (A) Immunoblot of Bcl-xL in SAOS-2 cell lysates that were incubated at pH 7.2, pH 7.4, or pH 7.6 for the indicated times. (B) Immunoblot of Bcl-xL from intact C33a, HTB-9, and SAOS-2 cells that were incubated in tissue culture medium at pH 6.5 or pH 7.3 and treated with 10 µM of monensin as indicated. (C) Immunoblot of purified bacterially synthesized wild-type Bcl-xL and Bcl-xL(N52A/N66A) that was incubated at pH 7.2, pH 7.4, or pH 7.6 for the indicated times. (D) SAOS-2 cells were treated with the indicated DNA-damaging agents. The percent of adherent cells with a pH above an arbitrarily chosen value of approximately pH 7.3 is indicated. (TIF) [file pbio.1001588.s003.tif]
