# Supplementary material for: Control of Cellular Bcl-xL Levels by Deamidation-Regulated Degradation
Source: PLoS Biol. 2013 Jun 25;11(6):e1001588. doi: 10.1371/journal.pbio.1001588 (PMC3692414; doi:10.1371/journal.pbio.1001588)
Supplement: Table S1 — Genbank accession numbers for Bcl-xL-like proteins. Genbank accession numbers for the proteins in Figures 1B and 2B are listed. (DOCX) [file pbio.1001588.s004.docx]

**Table S1. Genbank accession numbers for Bcl‑x_L_‑like proteins.** Genbank accession numbers for the proteins in Figure 1B and 1D are listed.

| **Species** | **Accession numbers** |
| --- | --- |
| *Suberites domuncula* | Genbank accession no. CAB97205 |
| *Geodia cydonium* | Genbank accession no. CAB97129 |
| *Oscarella carmela* | Translation of Genbank accession no. EC369942 |
| *Amphimedon queenslandica* | Translation of Genbank accession no. AC167698 |
| *Crassostrea gigas* | Genbank accession no. ACH42081 |
| *Aplysia californica* | Translation of Genbank accession no. EB211687 |
| *Tetraodon nigroviridis* | Genbank accession no. CAF93123 |
| *Gasterosteus aculeatus* | Translation of Genbank accession no. DW595641 |
| *Oncorhynchus mykiss* | Translation of Genbank accession no. BX913023 |
| *Salmo salar* | Translation of Genbank accession no. DY734223 |
| *Oncorhynchus nerka* | Translation of Genbank accession no. EV380352 |
| *Odontesthes bonariensis* | Genbank accession no. ACP19736 |
| *Gadus morhua* | Genbank accession no. ACZ62647 |
| *Osmerus mordax* | Genbank accession no. ACO09883 |
| *Dicentrarchus labrax* | Genbank accession no. CBN81010 |
| *Danio rerio* | Genbank accession no. NP571882 |
| *Rutilus rutilus* | Translation of Genbank accession no. EG548666 |
| *Pimephales promelas* | Translation of Genbank accession no. DT345680 |
| *Xenopus laevis* | Genbank accession no. NP001082147 |
| *Xenopus (Silurana) tropicalis* | Genbank accession no. NP001005428 |
| *Trachemys scripta elegans* | Genbank accession no. ACQ42286 |
| *Anolis carolinensis* | Genbank accession no. XP003229025 |
| *Lonchura striata* | Genbank accession no. AAY42379 |
| *Taeniopygia guttata* | Genbank accession no. XP002193122 |
| *Lagopus lagopus* | Genbank accession no. ADI93093 |
| *Gallus gallus* | Genbank accession no. CAA80657 |
| *Meleagris gallopavo* | Genbank accession no. XP003212117 |
| *Monodelphis domestica* | Genbank accession no. XP001362940 |
| *Sminthopsis crassicaudata* | Translation of Genbank accession no. EV533451 |
| *Cricetulus griseus* | Genbank accession no. ACC63896 |
| *Mus musculus* | Genbank accession no. AAC53459 |
| *Rattus norvegicus* | Genbank accession no. CAA57886 |
| *Spermophilus tridecemlineatus* | Genbank accession no. ABF56146 |
| *Ailuropoda melanoleuca* | Genbank accession no. XP002918329 |
| *Bos Taurus* | Genbank accession no. NP001070954 |
| *Canis lupus familiaris* | Genbank accession no. NP001003072 |
| *Equus caballus* | Genbank accession no. XP001499795 |
| *Felis catus* | Genbank accession no. NP001009228 |
| *Oryctolagus cuniculus* | Genbank accession no. NP001075604 |
| *Ovis aries* | Genbank accession no. NP001009226 |
| *Sus scrofa* | Genbank accession no. AAF33212 |
| *Lemur catta* | Genbank accession no. ADN43400 |
| *Saimiri boliviensis* | Uniprot accession no. E2IV77 |
| *Callithrix jacchus* | Genbank accession no. XP002747378 |
| *Macaca mulatta* | Genbank accession no. XP001110062 |
| *Pongo abelii* | Genbank accession no. XP002830236 |
| *Homo sapiens* | Genbank accession no. CAI12813 |
| *Trichoplax adhaerens* | Genbank accession no. XP002110477 |
| *S. purpuratus* | Genbank accession no. XP787479 |
| *Drosophila melanogaster* | Genbank accession no. AAF25955 |
| *Drosophila pseudoobscura* | Genbank accession no. XP001361966 |
| *Drosophila ananassae* | Genbank accession no. XP001961317 |
| *Drosophila yakuba* | Genbank accession no. XP002089347 |
| *Drosophila mojavensis* | Genbank accession no. XP002006331 |
| *Drosophila virilis* | Genbank accession no. XP002049521 |
| *Drosophila erecta* | Genbank accession no. XP001970756 |
| *Drosophila simulans* | Genbank accession no. XP002080303 |
| *Drosophila sechellia* | Genbank accession no. XP002032616 |
| *Drosophila grimshawi* | Genbank accession no. XP001985829 |
| *Drosophila willistoni* | Genbank accession no. XP002062746 |
