# Supplementary material for: Control of Cellular Bcl-xL Levels by Deamidation-Regulated Degradation
Source: PLoS Biol. 2013 Jun 25;11(6):e1001588. doi: 10.1371/journal.pbio.1001588 (PMC3692414; doi:10.1371/journal.pbio.1001588)
Supplement: Text S1 — Deamidation has no effect on the interaction of Bcl-xL with Bim or Bax. (DOCX) [file pbio.1001588.s005.docx]

**Text S1.**

Deamidation Has No Effect on the Interaction of Bcl-x_L_ with Bim or Bax

We first found that endogenous Bim binds the endogenous native and deamidated forms of Bcl-x_L_ equally well in both untreated and etoposide-treated cells (Figure S2A). We then examined binding in a cell line in which the expression of Bim is inducible and Bcl-x_L_ is constitutively overexpressed. The use of these cells allowed us to vary the ratio of Bim to Bcl-x_L_, such that we could examine the interaction between the two at limiting concentrations of Bim to determine if its affinity for each form of Bcl-x_L_ is the same. Additionally, because Bcl-x_L_ is overexpressed in these cells, any potential confounding effects of Bim-induced cell death were decreased. We found that even at limiting concentrations of Bim the ratio of bound deamidated to bound native Bcl-x_L_ remains proportional to the input of each form in both untreated cells (Figure S2B) and etoposide-treated cells (Figure S2C), indicating that the affinity of Bim for the deamidated and native forms of Bcl-x_L_ is the same. We were concerned that the deamidated forms of Bcl-x_L_ might only coimmunoprecipitate with Bim because the deamidated forms are bound either directly or indirectly to the native form of Bcl-x_L_. We addressed this possibility by examining the interaction between the deamidated and native forms of Bcl-x_L_ *in vivo*. When HA-tagged Bcl-x_L_ and untagged Bcl-x_L_ were coexpressed and the cells were treated with etoposide, the deamidated and native forms of both the HA-tagged and untagged Bcl-x_L_ coimmunoprecipitated with Bim; however, there was no interaction whatsoever between the HA-tagged and the untagged forms of Bcl-x_L_ (Figure S2D). Together, these findings demonstrate that deamidation has no effect on the interaction of Bcl-x_L_ with Bim. Our findings were similar when we examined the interaction of Bcl-x_L_ with Bax (Figure S2E).

**Supporting methods**

Plasmids and Bacterial Protein Synthesis

Bim-inducible SAOS-2 cells was described previously [16]. To generate purified bacterially synthesized Bcl-x_L_ proteins for direct assessment of the effects of pH on deamidation, pTri Ex-1.1-Bcl-x_L_(ΔTM) and pTri Ex-1.1-Bcl-x_L_(N52A/N66A/ΔTM) were constructed by ligation of the cDNA for codons 1 to 209 of each Bcl-x_L_ cDNA construct between the *Nco I* site and the *Bst1107I* site in the plasmid pTri Ex-1.1. The proteins encoded by these constructs were expressed in bacteria and purified using standard techniques. Assessment of the effect of pH on Bcl-x_L_ deamidation was performed as previously described [16]. We thank Dr. Eunhee Kim for providing pGFP-Bax plasmid.

Immunoblotting and Immunoprecipitation

The following antibodies were used: anti-Bim (AAP-330) from Stressgen for immunoblotting; anti-Bim (AM53) from Calbiochem and anti-GFP (G10362) from Invitrogen for immunoprecipitation.
