# Supplementary material for: Control of Cellular Bcl-xL Levels by Deamidation-Regulated Degradation
Source: PLoS Biol. 2013 Jun 25;11(6):e1001588. doi: 10.1371/journal.pbio.1001588 (PMC3692414; doi:10.1371/journal.pbio.1001588)
Supplement: Text S2 — DNA damage-induced Bcl-xL deamidation is regulated by changes in pH in the cell. (DOCX) [file pbio.1001588.s006.docx]

**Text S2.**

DNA Damage-Induced Bcl-x_L_ Deamidation Is Regulated by Changes in pH in the Cell

An increase in pH within the physiologic range is sufficient to increase Bcl-x_L_ deamidation in tumor cells lysates (Figure S3A). Using the sodium-proton exchanger monensin, we demonstrated that this is also true in intact tumor cells (Figure S3B). Deamidation is not dependent upon a deamidase because purified bacterially synthesized Bcl-x_L_ responds to increased pH in the same manner (Figure S3C). As has been demonstrated in other cell types [[37](#_ENREF_37),[65](#_ENREF_65),[67](#_ENREF_67)], we found that DNA-damaging agents increase the intracellular pH of SAOS-2 cells (Figure S3D), C33a cells, and HTB-9 cells (data not shown); however, we found that the increase the pH could only be detected in a subpopulation of the cells. The finding that the increase only occurs in a subpopulation of cells at a given point in time is consistent with the finding that deamidation of Bcl-x_L_ occurs over several days [[16](#_ENREF_16)]*.* Importantly, these cells lose their adherence in the later stages of apoptosis, therefore, we only assessed the pH of those cells that remained adherent in order to maximize the fraction of cells in the earlier stages of apoptosis in the assay.
